# Identification of the metabolic remodeling profile in the early-stage of myocardial ischemia and the contributory role of mitochondrion

**DOI:** 10.1080/21655979.2022.2068882

**Published:** 2022-04-26

**Authors:** Jun He, Qian Liu, Jie Wang, Fangjing Xu, Yucheng Fan, Ruhua He, Ru Yan, Li Zhu

**Affiliations:** a Department of Cardiovascular Internal Medicine, General Hospital of Ningxia Medical University, Yinchuan, Ningxia, People’s Republic of China; b School of Clinical Medicine, Ningxia Medical University, Yinchuan, Ningxia, People’s Republic of China; c School of Basic Medicine, Ningxia Medical University, Yinchuan, Ningxia, People’s Republic of China; d Department of Radiology, General Hospital of Ningxia Medical University, Yinchuan, Ningxia, People’s Republic of China

**Keywords:** Acute myocardial ischemia, mitochondria, metabolomics, myocardial remodeling, LC-MS/MS

## Abstract

Cardiac remodeling is the primary pathological feature of chronic heart failure. Prompt inhibition of remodeling in acute coronary syndrome has been a standard procedure, but the morbidity and mortality are still high. Exploring the characteristics of ischemia in much earlier stages and identifying its biomarkers are essential for introducing novel mechanisms and therapeutic strategies. Metabolic and structural remodeling of mitochondrion is identified to play key roles in ischemic heart disease. The mitochondrial metabolic features in early ischemia have not previously been described. In the present study, we established a mouse heart in early ischemia and explored the mitochondrial metabolic profile using metabolomics analysis. We also discussed the role of mitochondrion in the global cardiac metabolism. Transmission electron microscopy revealed that mitochondrial structural injury was invoked at 8 minutes post-coronary occlusion. In total, 75 metabolites in myocardium and 26 in mitochondria were screened out. About 23% of the differentiated metabolites in mitochondria overlapped with the differentiated metabolites in myocardium; Total 81% of the perturbed metabolic pathway in mitochondria overlapped with the perturbed pathway in myocardium, and these pathways accounted for 50% of the perturbed pathway in myocardium. Purine metabolism was striking and mechanically important. In conclusion, in the early ischemia, myocardium exacerbated metabolic remodeling. Mitochondrion was a contributor to the myocardial metabolic disorder. Purine metabolism may be a potential biomarker for early ischemia diagnosis. Our study introduced a perspective for prompt identification of ischemia.

## Graphical Abstract

**Figure:**
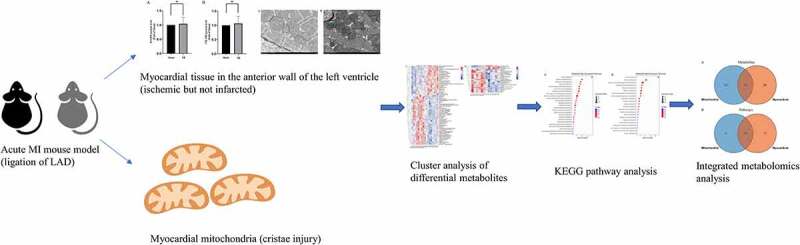

## 1. Introduction

Severe and persistent myocardial ischemia (MI) causes sudden decrease and even interruption of blood/oxygen supply in cardiomyocytes [1]. Fatal events, such as myocardial infarction, ventricular tachyarrhythmia, and even death often burst under these circumstances [2]. Interventional coronary reperfusion strategies are widely adopted to treat acute myocardial infarction, and initiation of cardiac remodeling treatment within 24 hours of hospitalization has been a standard procedure in the management of acute coronary syndrome, but the morbidity and mortality due to acute myocardial infarction and ischemic cardiomyopathy are still high [3–6].The identification of MI onset is crucial for timely intervention, subsequently helpful for reducing these adverse events. One characteristic of the chronic stage of ischemic heart disease is maladaptive myocardial remodeling [7], manifested as cardiomyocyte hypertrophy and death, extracellular matrix deposition (including fibrosis), and immune and inflammation injuries [8]. Although mounting studies focused on the underlying mechanisms of this pathological process, there are still gaps in our understanding of myocardial remodeling, and there are no effective strategies to reverse this process. This implies that cardiac remodeling maybe invoked much earlier, or the underlying mechanism is far beyond our certainty. In-depth profiling the molecular details and identifying biomarkers of early-staged MI may accelerate the diagnosis of cardiac remodeling, subsequently initiate the treatment sooner.

Changes in cardiac metabolism are causative in cardiac remodeling. However, the mechanisms of how these changes affect tissue form and function are unclear [9]. Mitochondrion is the ‘energy house’ in cells [10,11]. The high ATP demand of heart is fulfilled primarily by oxidative phosphorylation in mitochondrion, which contributes to about 95% ATP required by heart [9,11]. Mitochondrion homeostasis is crucial to cardiac metabolism and contraction [12]. Studies have shown that mitochondrial structure and metabolism remodeling are key characteristics of MI [7]. For instance, mitochondrial apoptosis and disrupted fission and fusion exacerbated cardiac ischemia-reperfusion injury in mouse [13,14]; disturbed mitochondrial dynamics was involved in cardiac microcirculation in ischemia-reperfusion injury and myocardial infarction [15–19]. Thousands of studies have presented mitochondrial metabolism profiles in ischemia-reperfusion injury [20–24] and acute myocardial infraction hearts, and have screened thousands of potential biomarkers of these processes [25–28]. However, report about mitochondrial metabolic characteristic in the early-stage of MI is lacking.

In the current study, we aimed to screen out potential metabolites in mitochondria and myocardium, and discuss the role of mitochondrion in global myocardial metabolism using liquid chromatography-mass spectrometry/mass spectrometry (LC-MS/MS). We hypothesized that mitochondria metabolism disturbance of the early-staged ischemic myocardium is inevitable and distinctive, subsequently contributing to potential cardiac metabolic disorder. Our work would be benefit for elucidating the characteristic and novel remodeling mechanism of the acute MI targeting mitochondrion.

## 2. Materials and methods

### 2.1. Experimental animals and groups

Eight-week-old male C57 BL/ 6 N mice, weighing 20–25 g, were purchased from Beijing Vital River Laboratory Animal Technology Co. Ltd. The animals were maintained in a specific pathogen-free colony of the Laboratory Animal Center of Ningxia Medical University with an indoor temperature of 22 ± 2°C, relative humidity of 40–60%, and 12: 12 hour light and dark cycle. The mice had ad libitum access to standard mouse chow and tap water. After one week of adaptive feeding, 24 animals were randomized into two groups; 12 were treated with LAD ligation (acute MI group) and 12 were treated with sham operation (Sham group). In each group, 6 mice were analyzed for heart tissue LC-MS/MS study, the other 6 were analyzed for mitochondria LC-MS/MS study (n = 6 per group). All experiments and protocols complied with the guidelines of the National Institutes of Health, Animal Care and Use Committee and were approved by the Ethics Review Committee of the General Hospital of Ningxia Medical University (ethical number 2016–038, 2020–101, Supplementary Materials).

### 2.2. Acute MI model establishment

The acute MI mice model was established by ligation of LAD artery as previously described [29–33]. 2% isoflurane (RWD Life Science, Shenzhen, China) was used for anesthesia at an airflow rate of 0.8–1.0 L /min. The effectiveness of anesthesia was defined as the disappearance of corneal and toe reflexes. After disinfecting the surgical area, the left chest was opened with a skin incision (1–2 cm) between the 3rd and 4th intercostal space in order to expose the heart. The pericardium was separated, the heart was exteriorized, and the LAD coronary artery was quickly ligated using a 6.0 prolene suture at approximately 1 mm distal to the left atrial appendage and 2 mm in width and depth to induce acute MI. The ST-segment elevation on electrocardiogram (ECG) (Labchart System, AD Instruments, USA) was used to confirm the successful occlusion of the LAD coronary artery. The heart was immediately placed back into the intrathoracic space once the ligation was completed. The chest cavity was then closed. The surgical operation in the Sham group was conducted in a similar manner without LAD artery ligation. After ligation, the animals were euthanized with 5% isoflurane. Cardiac tissues from the anterior wall of the left ventricular were cut immediately for analyses.

### 2.3. Serum D-lactate dehydrogenase (D-LDH) and creatine kinase isoenzyme-MB (CK-MB) measurement by enzyme-linked immunosorbent assay (ELISA)

After 15 min of ligation, whole blood was collected in serum separator tubes by eyes puncture. The blood was centrifuged at 4000 rpm for 20 min at room temperature, and serum was remained for assay. ELISA kits of D-LDH (Ruixinbio, RX203251M, Quanzhou, China) and CK-MB (Ruixinbio, RX201007M, Quanzhou, China) were used for detecting the enzymes as previously described [34,35]. The absorbance was measured at 450 nm. n = 6 per group. Samples were in triplicate.

### 2.4. Observation of the myocardium ultrastructure using transmission electron microscopy (TEM)

Cardiac tissues from the anterior wall of the left ventricular at LAD ligation of 8 min, 10 min, 12 min, 15 min and 20 min were collected for TEM observation. The freshly obtained myocardium (1 mm^3^) was rinsed with ice-cold PBS, and the buffer was removed using a clean filter paper. The tissue was fixed with 2% glutaraldehyde, washed thrice with 0.1 M dimethyl sodium arsenate, fixed with 4% osmic acid, rinsed once more in 0.1 M dimethyl sodium arsenate, and dehydrated using an alcohol gradient (30%–50%–70%). All the above processes were performed at 4°C. Next, the tissue was infiltrated with propylene oxide, fixed, and embedded in epoxy resin at 60°C for 48 h. Ultrathin sections of the embedded tissues were stained with 2% uranium acetate and lead citrate and then observed and imaged using TEM (Hitachi HT-7800, Tokyo, Japan).

### 2.5. Mitochondrial isolation

Mitochondria were isolated from the 15-min ischemic myocardium using a mitochondrial isolation kit (KeyGEN, KGA827, Jiangsu, China) as previously reported [36]. Procedure modification was made according to the manufacture’s instruction. Briefly, the freshly collected cardiac tissue (40–60 mg) of the anterior wall was immediately placed on ice, rinsed with cold saline, and dried by a clean filter paper. The tissue was transferred into a 2 mL glass homogenizer and cut into small pieces. Pre-cooled lysis buffer was added into the homogenizer until the cumulative volume was six times of the tissue volume. The tissue was ground 20 times at 4°C. The homogenate was transferred into a clean centrifuge tube containing 0.1 mL medium buffer (2 M sucrose), mixed gently, and centrifuged twice at 1,200 × g for 5 min at 4°C. The supernatant was collected and centrifuged at 7,000 × g for 10 min at 4°C. After discarding the supernatant, 0.15 mL of suspension buffer was added to the tube and centrifuged at 9,500 × g for 5 min at 4°C. The precipitate contained the isolated mitochondria. A portion of the purified mitochondria was used for determining the mitochondrial membrane potential immediately, and the rest was stored at −80°C for metabolomics analysis.

### 2.6. Measurement of mitochondrial membrane potential (MMP)

The JC-1 fluorescent probe detection kit (Beyotime, C2006, Shanghai, China) was used to determine the MMP according to the manufacturer’s instructions [37]. Briefly, JC-1 working solution was added to the freshly extracted mitochondria and mixed. The mixture was then added into a 96-well plate. The readings were measured using a fluorescence microplate reader (VICTOR Nivo, PerkinElmer, Waltham, Massachusetts, USA) at 485 nm excitation and 590 nm emission.

### 2.7. Untargeted metabolomics analysis by liquid chromatography-mass spectrometry/ mass spectrometry (LC-MS/MS)

#### 2.7.1. Metabolite extraction

Same volume of myocardium and mitochondrion from the ligated and sham hearts were extracted to prepare quality control (QC) samples. One QC sample was inserted into each 6-sample set for testing according to the following process: 300 *μ*L of pre-cooled extraction solvent (methanol: water, 80: 20, v/v) was added to approximately 30 mg of cardiac tissues, grounded for 2 min (50 Hz, −20°C), maintained at −20°C for 30 min, and centrifuged at 12,000 × g for 15 min at 4°C. Supernatant (250 *μ*L) was pipetted into a 1.5 mL centrifuge tube and dried using vacuum; 100 *μ*L of acetonitrile: water (90: 10, v/v) solution was added to the tube, followed by 5 min vortex, and centrifuged at 12,000 × g for 15 min at 4°C. Then, 1 *μ*L of supernatant was used for LC-MS metabolomics analysis. Pre-cooled extraction solvent (500 *μ*L, methanol: water = 80: 20, v/v) was added to freshly isolated mitochondria, crushed using ultrasonic cell crushing apparatus (20 kHz, 1 min) in ice water bath, maintained at −20°C for 30 min, and centrifuged at 12,000 × g for 15 min at 4°C. Supernatant (400 *μ*L) was transferred into a 1.5 mL centrifuge tube and dried using vacuum. One hundred microliters of acetonitrile: water (90: 10, v/v) solution was added in the tube, reconstituted via vortex for 5 min, and centrifuged at 12,000 × g for 15 min at 4°C. Then, 1 *μ*L of supernatant was used for metabolomics analysis.

#### 2.7.2. Liquid chromatography-mass spectrometry/ mass spectrometry (LC-MS/MS) analysis

Ultra high performance liquid chromatography (UHPLC, 1290 Infinity LC, Agilent Technologies, Germany) was used for LC-MS/MS analysis. The target compounds were isolated on a Waters ACQUITY UPLC BEH AMIDE column (2.1 * 100 mm, 1.7 *μ*m). The phase A of HPLC was water phase, containing 25 mmol/L ammonium acetate and 25 mmol/L ammonia water, and the phase B was acetonitrile. Gradient elution: 0–0.5 min, 95% B; 0.5–7 min, 95%–65% B; 7–8 min, 65%–40% B; 8–9 min, 40% B; 9–9.1 min, 40%–95% B; 9.1–12 min, 95% B. Mobile phase flow rate: 0.5 mL/min, column temperature: 30°C, sample tray temperature: 4°C, injection volume: 3 *μ*L.

The combined quaternary Orbitrap mass spectrometer (Q Exactive Orbitrap, Thermo Fisher Scientific, USA) based on the LC-MS/MS system was controlled by control software (Xcalibur, version 4.0.27, Thermo, USA) for primary and secondary mass spectrometric data acquisition. The detailed parameters were as follows. Sheath gas flow rate was 45Arb, aux gas flow rate was 15Arb, capillary temperature was 400°C, full MS resolution was 70,000, MS/MS resolution was 17,500, collision energy was 10/30/60 in NCE mode, spray voltage was 4.0 kV (positive) or −3.6 kV (negative).

#### 2.7.3. Data processing

The original data was converted into the MZXML format using ProteoWizard. The self-written R program package (kernel is XCMS) was used for peak recognition, peak extraction, peak alignment, and integration, then matched with the self-built secondary mass spectrometry database for material annotation. The cutoff value of the algorithm scoring was set to 0.3. The processed data were normalized and imported into the SIMCA-P + 14.0 software package (Umetrics, Umeå, Sweden) for unsupervised principal component analysis (PCA) and supervised orthogonal partial least squares discriminant analysis (OPLS-DA). To prevent over fitting of the model, the method of seven cycle interaction validation and 200 response ranking tests were used to examine the quality of the model. Variable importance in projection (VIP) scores in OPLS-DA analysis was calculated to indicate their contributions to grouping. In order to show the expression levels intuitively, the metabolites with VIP score>1.0 were further subjected to univariate level fold change (FC) analysis and Student’s *t*-test. The MetaboAnalyst platform based on the Kyoto Encyclopedia of Genes and Genomes (KEGG) Pathway Database was adopted for metabolic pathway enrichment analysis [38]. For all analyses, *P* values < 0.05 and | FC | > 1.5 were considered statistically significant.

### 2.8. Statistical analysis

SPSS 23.0 and GraphPad Prism 9.0 were used for statistical analysis. Data were depicted as mean ± SEM (standard error of the mean) and analyzed using Student’s *t*-test. The significance level was set at α = 0.05 and *P* < 0.05.

## 3. Results

Mitochondrion metabolism characteristic in the early-stage of MI remains unclear. In the current study, we aimed to identify potential metabolite in mitochondria and discuss its role in global myocardial metabolism by using a mimic animal via LC-MS/MS detection and bioinformatics analysis. Our goal was to demonstrate the characteristic and novel remodeling mechanism of the acute MI by targeting mitochondrion. We hypothesized that mitochondria metabolism disturbance is distinctive, subsequently contributing to potential cardiac metabolic disorder in the early-staged ischemia.

### 3.1. ECG

**Figure 1.**
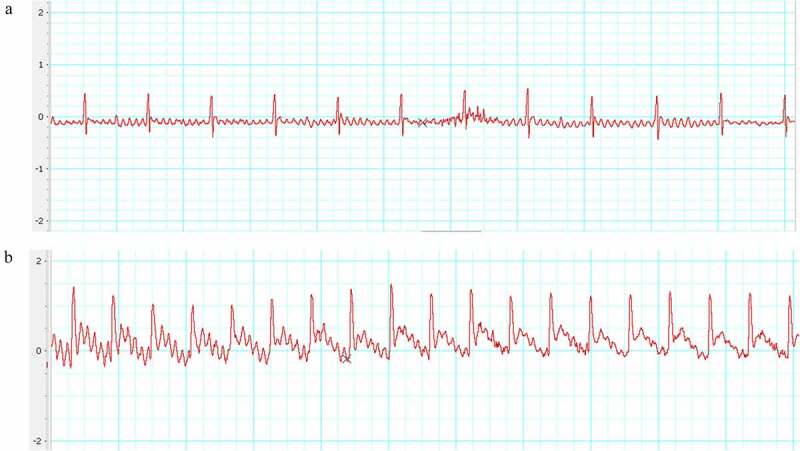
Electrocardiogram (ECG). (a) Before ligation; (b) After ligation. The ST-segment elevated dramatically than that of before ligation.

The ST-segment on ECG elevated 0.25–0.5 mV right after the ligation of LAD compared with the baseline (Figure 1). This indicated the LAD artery was successfully occluded.

### 3.2. D-LDH and CK-MB levels in the acute MI group

**Figure 2.**
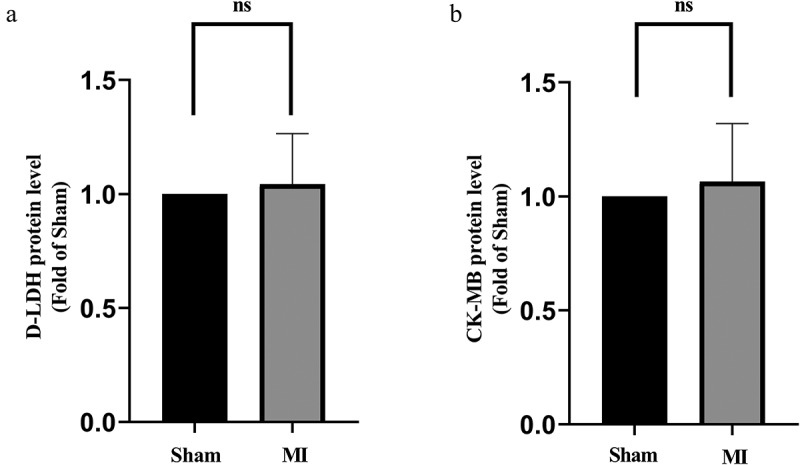
D-LDH and CK-MB levels in the acute MI group. (a) Comparison of D-LDH between the two groups; (b) Comparison of CK-MB between the two groups. No differences of D-LDH and CK-MB levels between the groups.

The D-LDH and CK-MB measurements are shown in Figure 2. D-LDH and CK-MB levels did not change significantly in the acute MI group compared to the Sham. This indicated that 15-min ligation did not induce detectable death of cardiomyocyte.

### 3.3. Myocardium morphology in the acute MI group detected by transmission electron microscopy (TEM)

**Figure 3.**
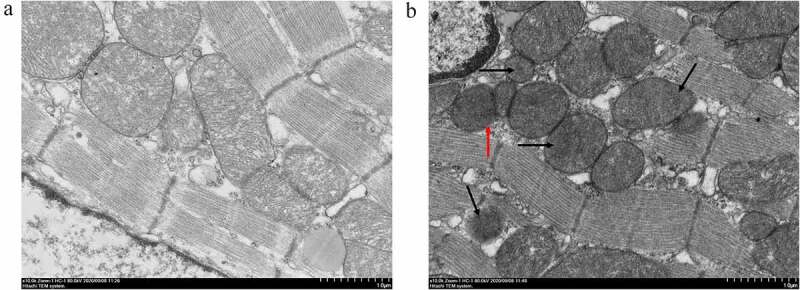
Mitochondrion morphology observed by TEM. The ultrastructure changes of the 15 min-ligation mitochondria are depicted in Figure 3. (a) Sham group; (b) acute MI group: dissolution of the outer membrane (red arrow), the ruptured or disappeared cristae (black arrow). The magnification is 10 000. Scale bar = 1 *μ*m.

The ultrastructure changes of 15 min-ligation myocardium are depicted in Figure 3 (× 10 000). The Sham group showed normal structure and architecture, with visible inner and outer membranes and regular arrangement of cristae in mitochondria (Figure 3a). The acute MI group showed partial dissolution of the mitochondrial outer membrane (Figure 3b, red arrow), and the cristae were ruptured or disappeared (Figure 3b, black arrow). The degree of abnormality was similar among ligation of 8 min, 10 min, 12 min, 20 min (Supplement Figure S1, × 10 000) and 15 min. The cardiomyocytes in both groups presented complete cell membrane and sarcomeres, well-arranged filaments, intact nuclear membrane, and uniform chromatin (Supplement Figure S2, × 1 500).

### 3.4. Mitochondrion membrane potential (MMP) level in acute MI heart

**Figure 4.**
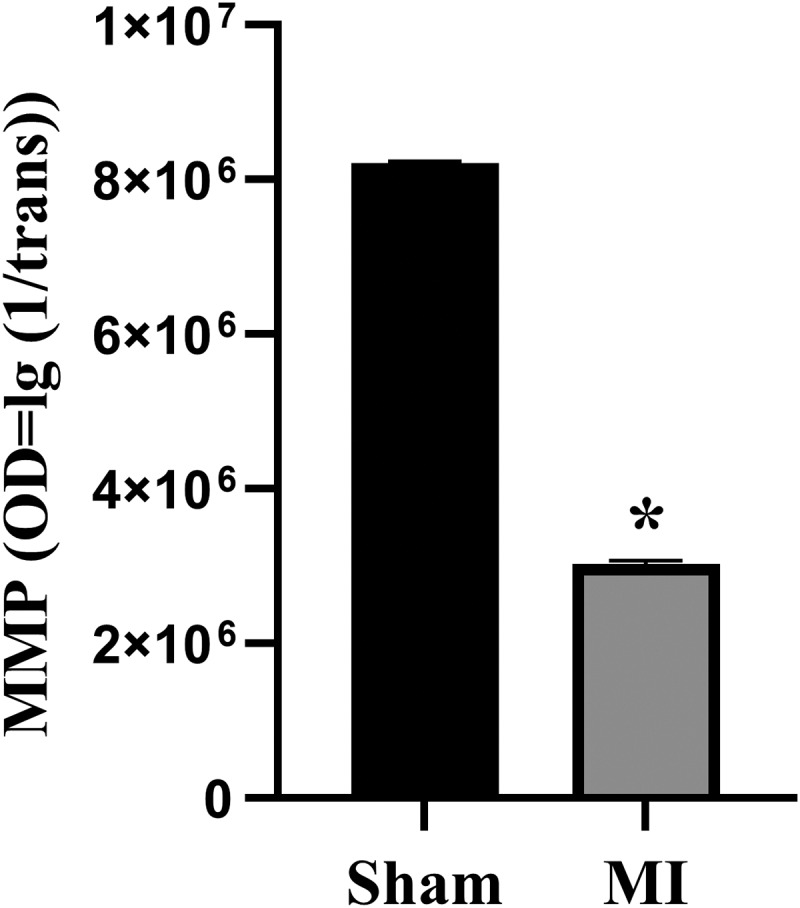
Measurements of mitochondrial membrane potential (MMP) levels. *P < 0.05, compared with the Sham group.

The acute MI group showed significant reduction in MMP compared with the Sham (*P* < 0.05, Figure 4). This showed mitochondrial dysfunction occurred due to the 15-min occlusion.

The above data suggested that a mouse model of early MI was established successfully.

### 3.5. Multivariate statistical analysis of metabolic profiles of myocardial tissue and mitochondria in acute MI mice

**Figure 5.**
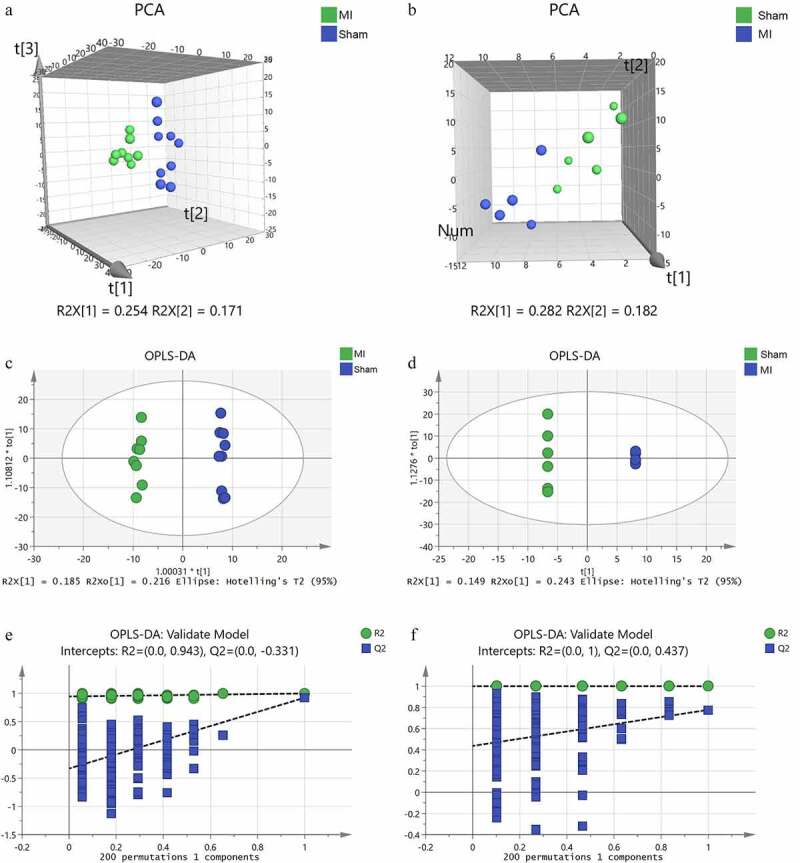
Multivariate statistical analysis of metabolic profiling of myocardial tissue and mitochondria in acute MI mice. Principal component analysis (PCA) score plot in myocardium (a) and mitochondria (b); orthogonal partial least squares discriminant analysis (OPLS-DA) score plot in myocardium (c) and mitochondria (d); statistical validation of the established OPLS-DA model with permutation analysis in myocardium (e) and mitochondria (f).

Preliminarily, PCA analysis was used to cluster the groups. For myocardium, there was a noticeable discrimination between the groups (R^2^X = 0.534, Q^2^ = 0.257, Figure 5a). Mitochondria also exhibited a reasonable separation between the two groups (R^2^X = 0.463, Q^2^ = 0.139, Figure 5b). Subsequently, the entire dataset was further identified using a supervised OPLS-DA. The OPLS-DA score plot is shown in Figure 5(c,d). Compared with the PCA, the OPLS-DA model showed advanced performance for clustering and the differences between the groups were further confirmed. The validity of the OPLS-DA model is shown in Figure 5(e,f). The results suggested that the stability and reproducibility of these analysis models were sufficient for differentiating the metabolite contents in the acute MI group and the control.

### 3.6. Identification of differential metabolites

**Figure 6.**
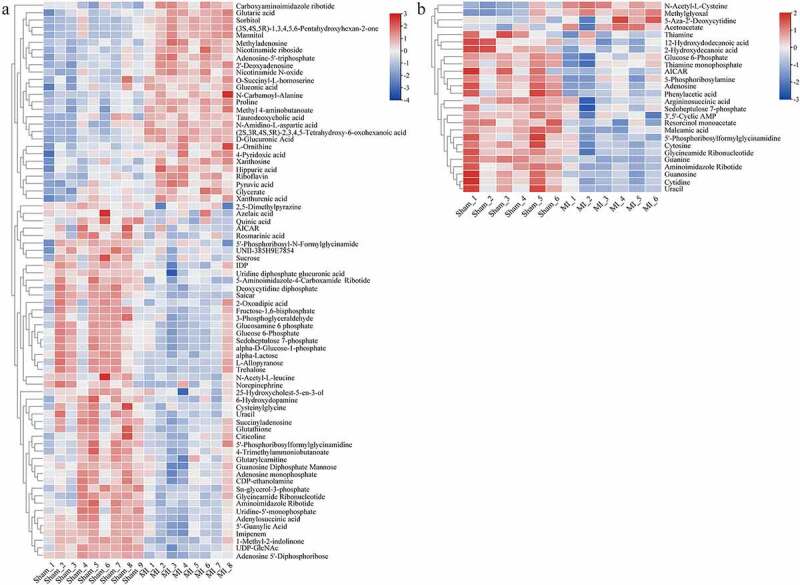
Hierarchical cluster analysis heat map of differential metabolites between groups in the myocardium (a) and mitochondria (b). Red indicates up-regulation, and blue indicates down-regulation. The columns and rows represent experimental samples and metabolites, respectively.

We screened the metabolites that contributed to the clusters using the VIP scores derived from the PCA and OPLD-DA analysis. The screening condition was VIP score > 1.0, *P* < 0.05, and | FC | > 1.5. In total, 75 differential metabolites in myocardium (Table 1) and 26 in mitochondria (Table 2) were identified between the two groups. Distinct clustering between the groups both in myocardium and mitochondria is depicted as a hierarchical clustering heat map (Figure 6(a,b)). The result indicated that these metabolites were potentially capable of distinguishing the acute MI group from the Sham group.

**Table 1.** List of differentially expressed metabolites in the myocardium

PubChem Name	Fold Change	*P* value	*P* (corr)	VIP score
Proline	1.5412	3.86E-06	−0.88871	1.74921
Sorbitol	4.5844	6.86E-05	−0.81761	1.59432
Nicotinamide riboside	2.5675	8.47E-05	−0.81341	1.6237
2’-Deoxyadenosine	1.6636	0.000185	−0.79057	1.58888
(2S,3 R,4S,5 R)-2,3,4,5-Tetrahydroxy-6-oxohexanoic acid	2.3733	0.000139	−0.78918	1.57752
(3S,4S,5 R)-1,3,4,5,6-Pentahydroxyhexan-2-one	4.4852	0.000282	−0.77913	1.52875
Adenosine-5’-triphosphate	2.2881	0.000462	−0.77111	1.52448
D-Glucuronic Acid	2.2013	0.000351	−0.76531	1.52899
Methyladenosine	1.999	0.000661	−0.75994	1.53626
Mannitol	4.7074	0.000471	−0.75921	1.48961
Methyl 4-aminobutanoate	1.5107	0.000976	−0.73087	1.46442
Glutaric acid	2.1317	0.001009	−0.72905	1.44395
Xanthosine	1.5674	0.003	−0.67263	1.30037
Riboflavin	1.5183	0.005976	−0.66483	1.31348
Nicotinamide N-oxide	1.5933	0.005297	−0.6542	1.3948
Hippuric acid	2.0497	0.006669	−0.6475	1.28647
N-Amidino-L-aspartic acid	3.0907	0.004751	−0.64211	1.26414
Carboxyaminoimidazole ribotide	1.9006	0.006763	−0.63905	1.2993
N-Carbamoyl-Alanine	1.8519	0.010606	−0.60242	1.18244
Glycerate	1.5204	0.011808	−0.59144	1.25651
Pyruvic acid	1.9496	0.018834	−0.59066	1.22256
Xanthurenic acid	1.6856	0.015463	−0.58943	1.17997
O-Succinyl-L-homoserine	1.5099	0.013738	−0.58313	1.13119
L-Ornithine	1.5098	0.015782	−0.57327	1.14204
Gluconic acid	1.5586	0.01638	−0.57224	1.11911
Taurodeoxycholic acid	2.0185	0.028568	−0.54223	1.11199
4-Pyridoxic acid	1.7705	0.026881	−0.53046	1.03618
25-Hydroxycholest-5-en-3-ol	0.50875	0.037556	0.507139	1.08709
Azelaic acid	0.65033	0.038202	0.51538	1.08174
2-Hydroxy-3-methylpentanedioic acid/2-Oxoadipic acid	0.65437	0.030624	0.518332	1.08478
N-Acetyl-L-leucine	0.33905	0.031132	0.52263	1.02254
Glutarylcarnitine	0.65429	0.029873	0.524366	1.14559
alpha-Lactose	0.072324	0.029482	0.524572	1.06297
N-{(2S)-1-[(Carboxymethyl)amino]-1-oxobutan-2-yl}-L-glutamine	0.60419	0.023834	0.542813	1.11771
Norepinephrine	0.65773	0.016057	0.572284	1.20696
2,5-Dimethylpyrazine	0.56438	0.015547	0.590615	1.25847
L-Allopyranose	0.46458	0.010139	0.596707	1.23312
Palatinose/Sucrose	0.65577	0.011183	0.599831	1.17403
AICAR	0.62266	0.01033	0.607074	1.25351
5’-Phosphoribosylformylglycinamidine	0.6066	0.009054	0.614037	1.21355
Trehalose	0.31178	0.007387	0.61927	1.26999
Quinic acid	0.60599	0.009021	0.624643	1.33058
Glycineamide Ribonucleotide	0.63645	0.00699	0.630657	1.23515
Sn-glycerol-3-phosphate	0.42848	0.008439	0.633728	1.24838
IDP	0.42028	0.005966	0.642071	1.30608
Fructose-1,6-bisphosphate	0.56145	0.003718	0.658876	1.34631
Adenosine monophosphate/2’-Deoxyguanosine 5’-Monophosphate	0.43617	0.004195	0.66999	1.29648
5’-Phosphoribosyl-N-Formylglycinamide	0.37942	0.00279	0.670704	1.31274
Uridine-5’-monophosphate	0.3857	0.003803	0.673328	1.31429
CDP-ethanolamine	0.62711	0.004014	0.675259	1.32088
Citicoline	0.66279	0.002654	0.689802	1.3365
Glucose?6-Phosphate	0.36119	0.002323	0.690311	1.40783
Aminoimidazole Ribotide	0.38899	0.001734	0.694361	1.34745
(2 R,3 R,4S,5 R)-2,3,4,5-Tetrahydroxy-6-oxohexyl dihydrogen phosphate	0.37173	0.001505	0.707942	1.44432
alpha-D-Glucose-1-phosphate	0.42245	0.001168	0.719134	1.47417
Succinyladenosine	0.47463	0.001376	0.723293	1.40372
Cysteinylglycine	0.64331	0.000828	0.723481	1.40289
Glucosamine 6-phosphate	0.5066	0.001187	0.724859	1.47289
Rosmarinic acid	0.53182	0.000774	0.736906	1.44131
6-Hydroxydopamine	0.62983	0.000763	0.740208	1.4663
4-Trimethylammoniobutanoate	0.64617	0.000513	0.748743	1.49054
Deoxycytidine diphosphate	0.48425	0.000514	0.749378	1.51249
Adenylosuccinic acid	0.10358	0.000372	0.765665	1.4817
Glutathione	0.61612	0.000268	0.781953	1.52574
Guanosine Diphosphate Mannose	0.35574	0.000219	0.791177	1.55046
3-Phosphoglyceraldehyde/Dihydroxyacetone phosphate	0.64779	0.000116	0.7942	1.56371
5’-Guanylic Acid	0.24286	0.000102	0.810701	1.58825
Saicar	0.31162	6.86E-05	0.813665	1.61634
Uracil	0.60938	6.55E-05	0.8159	1.63105
5-Aminoimidazole-4-Carboxamide? Ribotide	0.36423	2.27E-05	0.830498	1.62349
Imipenem	0.144	1.71E-05	0.84872	1.67532
UDP-GlcNAc	0.36854	2.66E-05	0.852852	1.65222
Adenosine 5’-Diphosphoribose	0.076901	1.17E-05	0.867379	1.69076
Uridine diphosphate glucuronic acid	0.35557	8.97E-06	0.869146	1.71013
1-Methyl-2-indolinone	0.29404	5.14E-06	0.874355	1.69211

**Table 2.** List of differentially expressed metabolites in mitochondria

PubChem Name	Fold Change	*P* value	VIP score	*P* (corr)
Guanine	0.32141	0.000108	1.83768	−0.90861
Cytidine	0.34279	0.026188	1.45118	−0.66294
Aminoimidazole Ribotide	0.34757	0.004086	1.63382	−0.78658
Guanosine	0.37653	0.006126	1.60848	−0.76458
12-Hydroxydodecanoic acid	0.38563	0.02757	1.40033	−0.65855
Cytosine	0.38656	0.010971	1.51331	−0.72851
5-Phosphoribosylamine	0.41281	0.018761	1.47253	−0.68999
Uracil	0.41963	0.013371	1.52737	−0.71504
Glycineamide Ribonucleotide	0.43421	0.006127	1.60899	−0.76466
2-Hydroxydecanoic acid	0.45438	0.035809	1.38763	−0.63491
3’,5’-Cyclic AMP	0.46725	0.010047	1.51955	−0.73442
Argininosuccinic acid	0.47335	0.026555	1.42955	−0.66176
Glucose 6-Phosphate	0.48313	0.001868	1.69358	−0.82276
Adenosine	0.51252	0.006006	1.60229	−0.76568
5’-Phosphoribosylformylglycinamidine	0.52988	0.025324	1.42294	−0.66578
Thiamine monophosphate	0.53703	0.043485	1.36121	−0.61615
{[(2 R,3S,4 R,5S)-3,4,5,6-Tetrahydroxy-6-(hydroxymethyl)oxan-2-yl]methoxy}phosphonic acid	0.54264	0.047841	1.35577	−0.60666
Phenylacetic acid	0.55089	0.010009	1.54329	−0.73465
AICAR	0.55954	0.037992	1.35359	−0.62923
Thiamine	0.58891	0.031914	1.35425	−0.64556
Maleamic acid	0.6605	0.00122	1.72477	−0.83979
Resorcinol monoacetate	0.66203	0.010565	1.47608	−0.73138
N-Acetyl-L-Cysteine	1.5574	0.00034	1.78634	0.881038
Methylglyoxal	1.5685	0.003986	1.64131	0.787843
5-Aza-2’-Deoxycytidine	1.5934	0.037113	1.32047	0.631844
Acetoacetate	1.5935	0.003818	1.60644	0.789853

### 3.7. Metabolic pathway enrichment analysis

**Figure 7.**
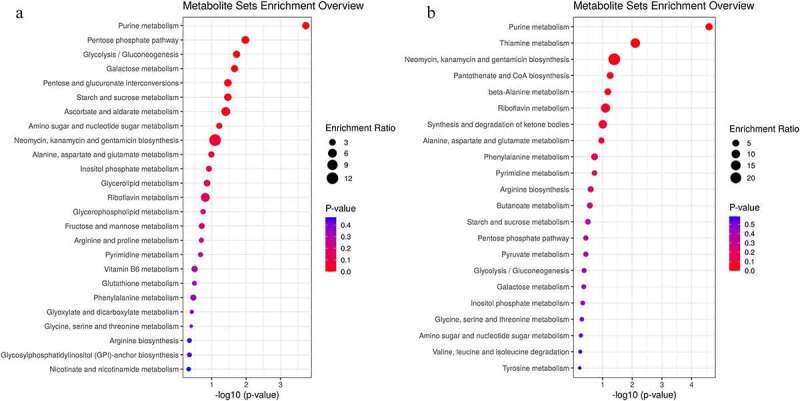
Enrichment analyses of common differential metabolites in the myocardium (a) and mitochondria (b) in Sham and acute MI groups after acute MI. Each bubble represents a metabolic pathway. The abscissa and size of the bubble represent the size of the influencing factor in the topological analysis. The larger the size, the larger the influencing factor. The ordinate and bubble color represent the *P*-value of enrichment analysis (considering the negative natural logarithm, i.e., −log10 (*P*-value)). The redder the color, the smaller the P-value, the greater the significance of the corresponding KEGG pathway enrichment.

We analyzed all the differential metabolites using the KEGG Pathway Database to identify the disturbed metabolic pathways due to the LAD occlusion. The results are depicted as bubble diagrams. As shown in Figure 7a, 34 disturbed pathways in the ischemic myocardium were identified, in other words, the differential metabolites mainly enriched in these pathways. These pathways are involved in purine and pentose phosphate metabolism, glycolysis/gluconeogenesis and galactose metabolism, pentose and glucuronate interconversions metabolism, starch and sucrose metabolism, and ascorbate and aldarate metabolism. Twenty-one perturbed pathways were identified in the ischemic mitochondrion (Figure 7b), the top three were purine metabolism, thiamine metabolism, and neomycin, kanamycin, and gentamicin biosynthesis.

### 3.8. Integrated analysis of enrichment pathway in the ischemic myocardium and mitochondrion

**Figure 8.**
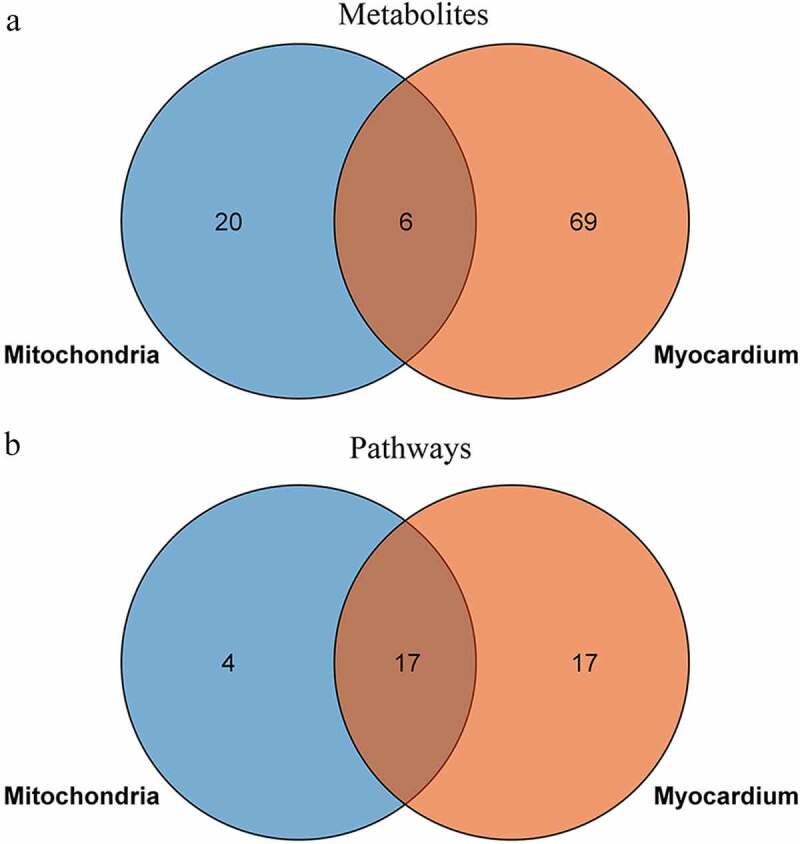
Venn diagram of perturbed metabolites (a) and metabolic pathways (b) in the myocardium and mitochondria after acute MI.

To explore the relationship between the ischemic myocardium and mitochondria metabolic disturbances, we used the KEGG pathway analysis as the carrier for mapping analysis of the disturbed pathways. The Venn diagram shows the shared and unique metabolites and pathways in the ischemic myocardium and mitochondria metabolomics (Figure 8(a,b)). Some commonly shared metabolites included aminoimidazole ribotide, AICAR (acadesine), uracil, glycineamide ribonucleotide, glucose-6-phosphate, and 5’-phosphoribosyl formylglycinamidine. The commonly shared pathways mainly included glycolysis/gluconeogenesis; pentose phosphate pathway; alanine, aspartate, and glutamate metabolism; purine and pyrimidine metabolism.

## 4. Discussion

Ischemic heart disease is a major illness and a leading cause of death worldwide [39]. Longer duration of ischemia usually leads to much more severe myocardial damage. Cardiomyocyte injury in myocardial infarction is irreversible as opposed to early myocardial ischemia [40]. Timely diagnosis and intervention of ischemia is critical for preventing heart from infarction, therefore life-saving. More explorations of the underlying molecular activities are essential to achieve earlier identification of MI. The present study aimed to explore the molecular details and identify potential biomarkers of early MI to flourish the experimental evidence for the diagnosis of early MI. We observed significant metabolism changes in mitochondrion and myocardial tissues. Purine metabolites were striking and purine metabolism was identified mechanistically important. With future replications, the identified biomarkers can potentially inform early detection of MI, subsequently advance the treatment of acute coronary syndrome.

Given the data of ECG (Figure 1) and D-LDH and CK-MB measurements (Figure 2), an animal model that was in the early-stage of MI was confirmed. The myocardium morphology observations (Figure 3 & Supplements) and MMP assay (Figure 4) presented mitochondrion structural impairment and dysfunction. These data indicated that mitochondrion was extremely sensitive to depletion of oxygen and apt to incur structural damage. Hence, targeting mitochondrion to explore the characteristic of early MI is an appropriate approach.

Metabolomics study based on LC-MS/MS analysis is capable of profiling metabolic characteristics and is powerful in determining trace concentrations of metabolites in samples, therefore, is an efficient way for exploring molecular activities in diseases. It has been applied as a classic and natural method in elucidating molecular changes and identifying candidate biomarkers of diseases [41–43]. In the current study, we screened out 75 differentiated metabolites in myocardium (Table 1) and 26 in mitochondria (Table 2) between the acute MI group and the sham group by PCA and OPLS-DA models (Figure 5, Figure 6). Purine metabolism disturbance was extremely prominent in the acute MI hearts. Among all the differentiated metabolites, almost 30% (21/75) in the ischemic myocardium and more than 40% (11/26) in the ischemic mitochondrion were nucleosides and nucleotides (Table 1 and 2). KEGG enrichment analyses revealed the perturbed pathway mainly included purine and pyrimidine metabolism, glycometabolism (e.g., glycolysis/gluconeogenesis, pentose phosphate, and galactose metabolism), amino acid metabolism (e.g., alanine, aspartate, and glutamate). Among them, purine metabolism pathway was the most significantly perturbed pathway, both in the ischemic myocardial tissue and mitochondria (Figure 7(a,b)). This implied that purine metabolism was more significant and meaningful for the energy production in early MI. Purine metabolism is the vital in ATP production. Studies have shown that purinergic signaling is closely related to cardiac energy metabolism [44]. An increase in energy expenditure results in an increase of purine nucleotides and their metabolites. Szabo et al [45] demonstrated that during hypoxia, the degradation products of adenosine and hypoxanthine are better energy sources than extracellular glucose. In cells, the utilization and transformation process of ATP is ATP–ADP–AMP–adenosine–hypoxanthine nucleoside–hypoxanthine–xanthine–uric acid. In our work, the down-regulated adenosine in ischemic mitochondria and up-regulated xanthine in ischemic myocardium were probably compensatory via the active purine metabolism.

Altered metabolites of saccharides and amino acids were minor in our study. Related metabolites accounted for 16% (12/75) and 12% (3/26) of the total differentiated molecules in myocardium and mitochondria respectively (Table 1 and 2). Under ischemic and hypoxic conditions, aerobic oxidation of glucose is restricted and glycolysis becomes an effective metabolic mode for energy providing [46,47]. Glucose is transported into cells and phosphorylated to produce glucose-6-phosphate. This irreversible reaction is the first rate-limiting step of glycolysis. Pyruvate, the final product of glycolysis, is catalyzed to lactic acid by lactate dehydrogenase (LDH); then, more NAD+ is produced. This process improves the ratio of NAD+/NADH and ATP generation [48]. In our study, up-regulated pyruvate and down-regulated glucose-6-phosphate indicated that anaerobic glycolysis probably enhanced during the sudden depletion of oxygen. Slight alteration of amino acids metabolism was observed in our model. For example, L-ornithine was up-regulated and argininosuccinic acid, an intermediate of ornithine cycle, was down-regulated in the ischemic hearts. L-ornithine is a key precursor for the synthesis of L-citrulline and L-arginine, as well as a downstream product of glutamic acid, which plays an important role in myocardial ischemia [49]. As a central part of the ornithine cycle, L-ornithine can detoxify harmful ammonia in human [50,51]. In the current study, the changed levels of ornithine and argininosuccinate acid may represent the disturbed amino acid transamination. This may be adaptive.

Importantly, almost no metabolite or pathway related to fatty acids was screened out between the two groups. That meant the heart maintained homeostasis of fatty acid metabolism during the short-term ischemia. Physiologically, fatty acids oxidation is the most efficient way for ATP production, and fatty acids function as major substrates. During the chronic stage of MI and pathological remodeling, the heart switches its substrate preference from fatty acids to glucose [52–54]; however, these changes in cardiac metabolism are inconsistent [9]. In pathological hypertrophy or heart failure, fatty acid up take and oxidation may be diminished [55–57], augmented [58] or unchanged [59]. Relevance about fatty acids metabolism disorder and myocardial ischemia has been explored recently. One study reported that fatty acids showed differential concentrations at 5 min post coronary occlusion compared to baseline in the serum of human. There was an increased use or a decreased synthesis of short and medium chain fatty acids, and a decreased use or an increased synthesis of long chain and very long chain fatty acids for beta-oxidation [40]. Sun et al [60] described an aberrant fatty acid metabolism in isoproterenol hydrochloride-induced acute myocardial infarction rats. By biochemical and targeted metabolomics analysis, the researchers found that free fatty acids (FFAs) alterations were significant in the plasma and myocardial tissues. They revealed that palmitic acid, oleic acid, linoleic acid and arachidonic acid were potentially the most relevant FFAs to inflammatory, apoptosis and necrosis in the infarcted heart. Another work showed that long-chain acyl-CoA and acylcarnitine increased in the mitochondria isolated from an ischemic area of the acute ischemia/reperfusion myocardium, in which long-chain acylcarnitines accumulation was harmful to mitochondria and induced inhibition of oxidative phosphorylation [61]. Obviously, the specific stages of MI that these studies focused on differed from our view, and the approaches that were used to induce animal model were mechanically different from which we adopted. Regardless, these evidences suggested that fatty acids respond differently to MI and may have important downstream effects, but details remain to be explored. In the present study, the minor disturbance of saccharides and amino acids metabolism and undisturbed fatty acids metabolism implied that fatty acids were the primary substrates which the heart relied on for energy production in early MI. Namely, the heart essentially used fatty acids as fuel to meet its ATP consumption almost without substrates switching during the initiation of MI.

Based on the above, either mitochondria or myocardium incurred significant metabolism remodeling in the early-stage of MI. In order to explore the role of mitochondrion in the global myocardial metabolism, we completed LC-MS/MS integrated analyses. The result showed that 23% (6/26) of the differentiated metabolites in mitochondria overlapped with those in myocardium (Figure 8a); however, 81% (17/21) of the disturbed pathways in mitochondria were involved in myocardium metabolism (Figure 8b). This indicated that alteration of mitochondrial metabolism was closely related to the myocardial metabolism remodeling in the early-staged ischemia. The findings demonstrated a significant contributory role of mitochondrion to the abundant metabolism remodeling in acute MI, and provided an evidence of mitochondrion may be potential target for acute MI intervention.

Several issues kept unaddressed. First, the study is observatory, further investigations are warranted to identify the differential metabolites’ downstream effects, and especially how the metabolites affect myocardial tissue form. Additionally, in terms of the morphology observation (Supplement Figure S1), it is difficult to make out the time course of mitochondrion metabolism alteration without biochemical and metabolomics supportive evidence. For example, mitochondrion is a dynamic system and thus the changes induced are transient and may be in the transition from fused to fission stage or vice versa. Hence, experiments are essential to get more supportive evidence to make a confirmatory change which is reliable in all occasion.

## 5. Conclusion

Our work discovered a novel metabolism remodeling. In the early-stage of MI, the cardiomyocytes relied on fatty acids as the primary substrate to meet energy demand via increased purine metabolism. Mitochondrion played an important contributory role in the global cardiac metabolism disturbance. This type of remodeling may be adaptive and temporary. Mitochondrion was vulnerable to oxygen depletion and subsequently presented deteriorated structure and function. Consideration of metabolism remodeling intervention targeting mitochondria in the initial stage of MI is reasonable. The present study was observatory and limited, further investigations are essential to explore more details and the underlying molecule activities.

## Supplementary Material

Supplemental Material
