# Supplementary material for: Identification of the metabolic remodeling profile in the early-stage of myocardial ischemia and the contributory role of mitochondrion
Source: Bioengineered. 2022 Apr 26;13(4):11106–21. doi: 10.1080/21655979.2022.2068882 (PMC9161979; doi:10.1080/21655979.2022.2068882)
Supplement: Supplemental Material [file KBIE_A_2068882_SM9856.zip › supplementary/editorial.pdf]

101

宁夏医科大学总医院科研伦理委员会
批 准 书

项目名称：缺氧条件下泛素连接酶 MuRF2 调控心肌能量代谢重构的机制研究	伦理编号：2020--101

项目负责人：何军	职称：主任医师	联系电话：13995273232	科室：心内科

负责研究单位：宁夏医科大学总医院

合作研究单位：无

研究时间： 2020 年 8 月至 2022 年 8 月

研究项目来源：

☑纵向研究（政府支持） □协会/基金会 □公司 □横向合作

□多中心研究 □自主 □其他

研究经费资助单位：宁夏科学技术厅

评审意见：

同意

☑ 符合伦理学要求，可以按照此方案进行试验。
□ 不符合伦理学要求，请修改后再报伦理委员会审查。
知情同意书：有□ 无□
获取知情同意书方法：适当□ 不适当□

专家签字：

方建群 0081

马辉 1232

[illegible] 019[illegible]

宁夏医科大学总医院科研伦理委员会

主任委员：（签章）

2020 年 1 月 16日

Scanned by CamScanner
