# Supplementary figures and images for: Identification of the metabolic remodeling profile in the early-stage of myocardial ischemia and the contributory role of mitochondrion
Source: Bioengineered. 2022 Apr 26;13(4):11106–21. doi: 10.1080/21655979.2022.2068882 (PMC9161979; doi:10.1080/21655979.2022.2068882)

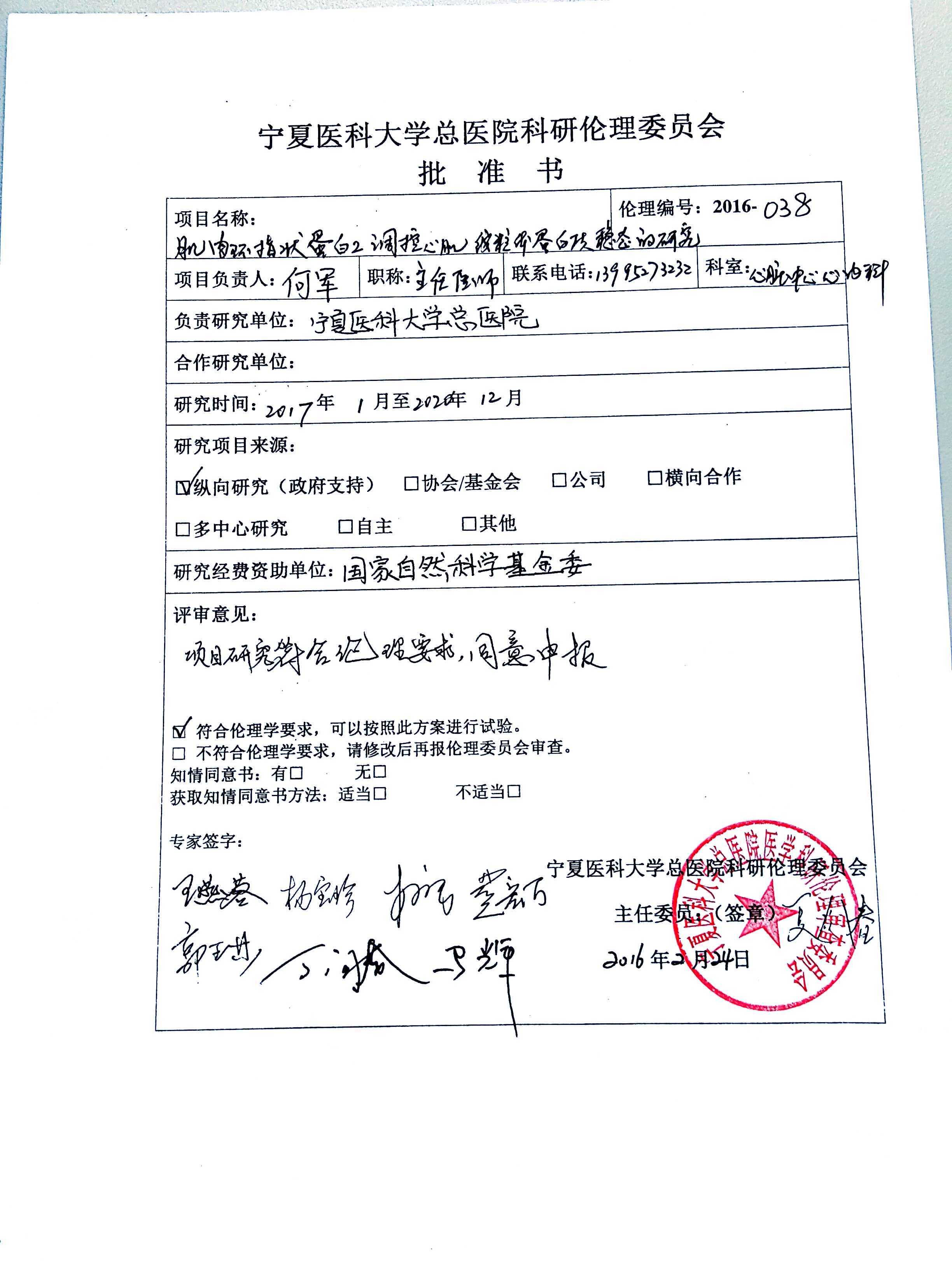

Supplement: Supplemental Material [file KBIE_A_2068882_SM9856.zip › supplementary/Ethical approval.jpg]

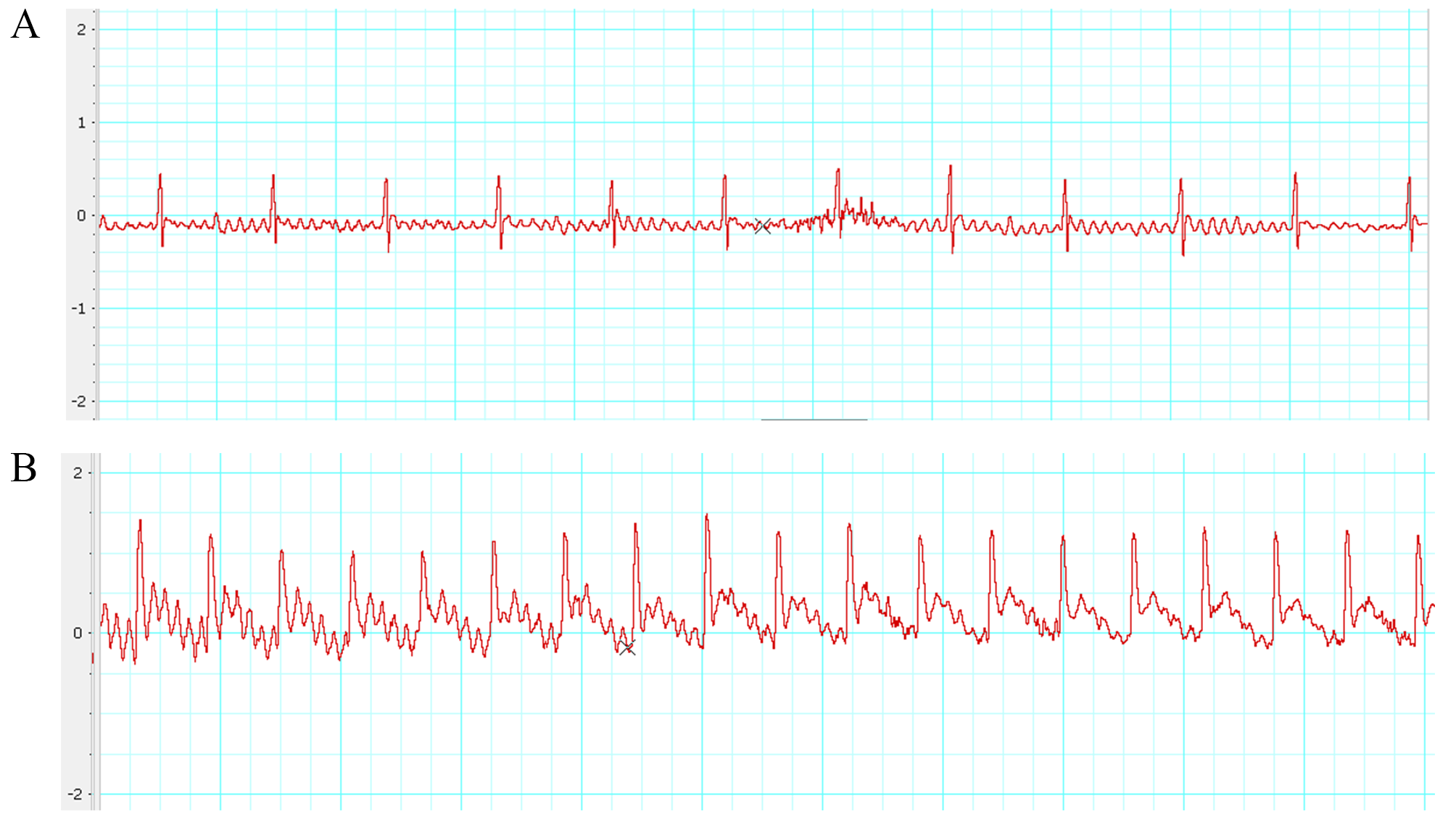

Supplement: Supplemental Material [file KBIE_A_2068882_SM9856.zip › supplementary/Supplementary Figure 1.tif]

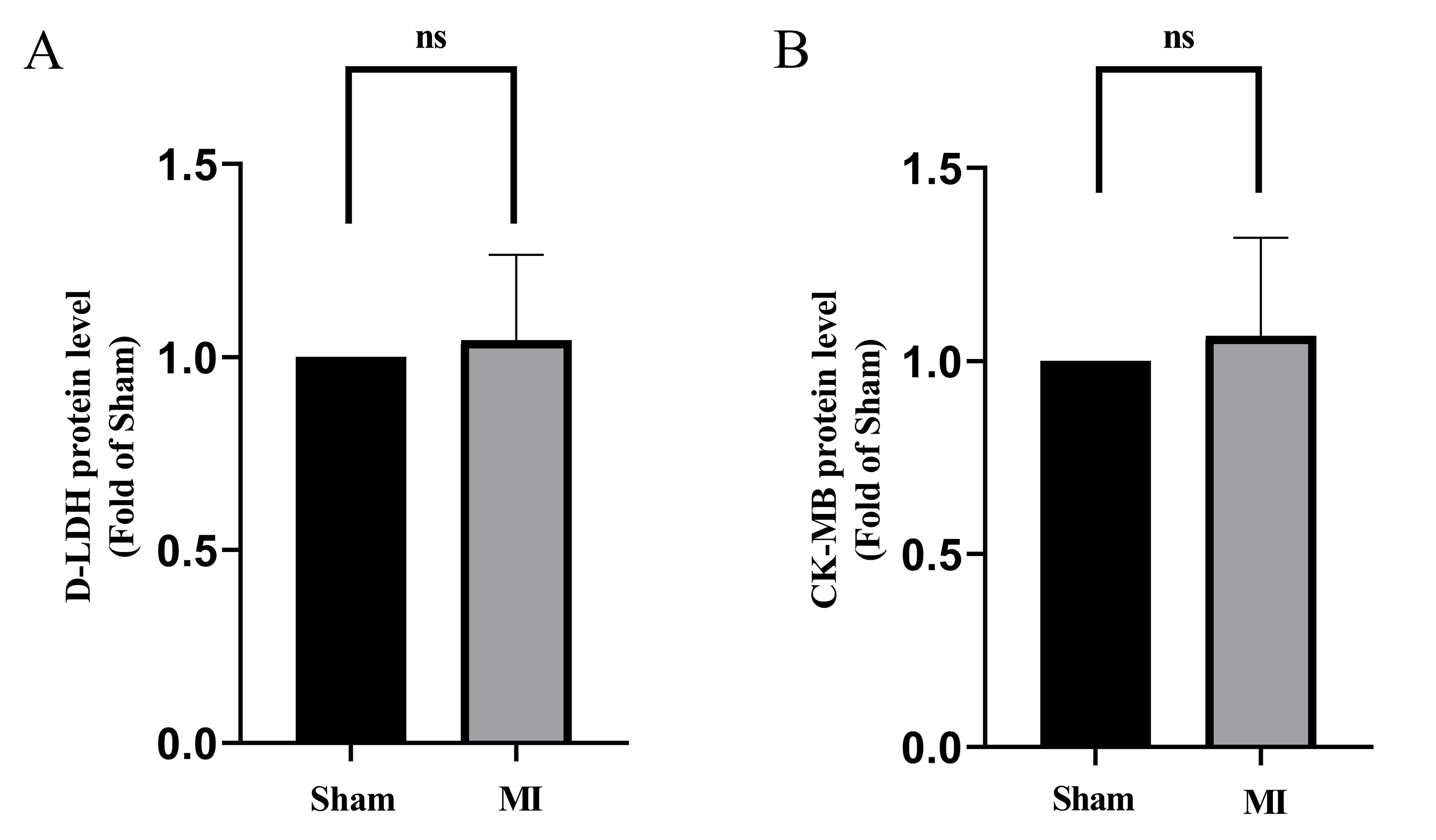

Supplement: Supplemental Material [file KBIE_A_2068882_SM9856.zip › supplementary/Supplementary Figure 2.tif]
